# Pharmacomicrobiomics in Pediatric Oncology: The Complex Interplay between Commonly Used Drugs and Gut Microbiome

**DOI:** 10.3390/ijms232315387

**Published:** 2022-12-06

**Authors:** Davide Leardini, Francesco Venturelli, Francesco Baccelli, Sara Cerasi, Edoardo Muratore, Patrizia Brigidi, Andrea Pession, Arcangelo Prete, Riccardo Masetti

**Affiliations:** 1 Pediatric Oncology and Hematology “Lalla Seràgnoli”, IRCCS Azienda Ospedaliero Universitaria di Bologna, 40138 Bologna, Italy; 2 Department of Experimental, Diagnostic and Specialty Medicine (DIMES), University of Bologna, 40126 Bologna, Italy; 3 Department of Medical and Surgical Sciences (DIMEC), University of Bologna, 40126 Bologna, Italy; 4 Pediatric Unit, IRCCS Azienda Ospedaliero Universitaria di Bologna, 40138 Bologna, Italy

**Keywords:** gut microbiota, oncology, pharmacomicrobiomics, chemotherapy, antibiotics, pediatrics

## Abstract

The gut microbiome (GM) has emerged in the last few years as a main character in several diseases. In pediatric oncological patients, GM has a role in promoting the disease, modulating the effectiveness of therapies, and determining the clinical outcomes. The therapeutic course for most pediatric cancer influences the GM due to dietary modifications and several administrated drugs, including chemotherapies, antibiotics and immunosuppressants. Interestingly, increasing evidence is uncovering a role of the GM on drug pharmacokinetics and pharmacodynamics, defining a bidirectional relationship. Indeed, the pediatric setting presents some contrasts with respect to the adult, since the GM undergoes a constant multifactorial evolution during childhood following external stimuli (such as diet modification during weaning). In this review, we aim to summarize the available evidence of pharmacomicrobiomics in pediatric oncology.

## 1. Introduction

**Figure 1:**
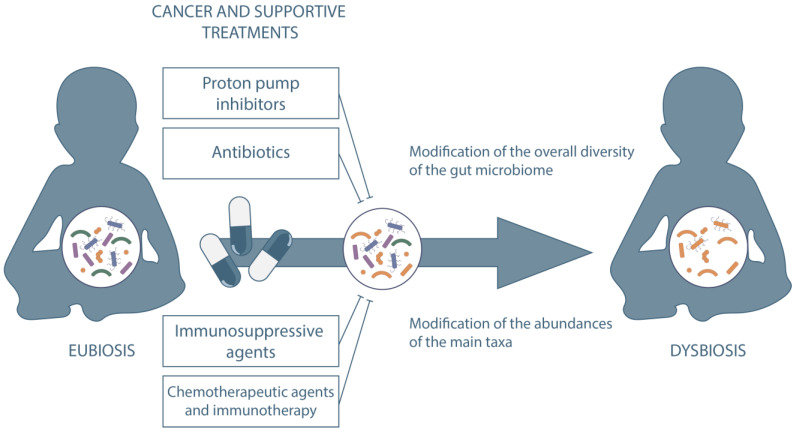
Impact on GM of drugs commonly used in clinical practice in pediatric patients with cancer.

The gut microbiome (GM) is considered a major determinant in human health with a pivotal role in the modulation of the immune system [1]. In oncology, the GM has been demonstrated to contribute in the pathogenesis of cancer [2,3] and to play a major role during anticancer therapy. GM dysbiosis, characterized by reduced ecosystem diversity, loss of health-related commensals and overgrowth of potentially pathogenic bacterial species, occurs frequently in cancer patients [2,4]. These GM modifications have been associated with clinical outcomes in cancer patients. For example, intestinal domination, defined as the presence of a single bacterial taxon comprising more than 30% of the entire GM, of Enterococcus is associated with increased mortality in the acute leukemia chemotherapy population [5]. Moreover, GM composition correlates with response and toxicity following anti-CD19 CAR T cell therapy [6] and anti-PD1 immunotherapy [7]. Most of the current literature addresses the role of the GM during hematopoietic stem cell transplantation (HSCT). The intestinal ecosystem undergoes a significant disruption during the procedure due to a combination of upsetting events [8,9]. In particular, intestinal mucosal barrier damage associated with the conditioning regimen, dietary alterations and the use of broad-spectrum antibiotics profoundly injures the GM [4,10,11,12]. The resulting alterations in the GM architecture are correlated with clinical outcomes. In recent years, new methodologies for studying the GM have extended our comprehension of the host-microbiome interaction, shedding light on bacterial functional properties such as metabolomics and metagenomics [13,14]. Drug-metabolizing activity of human gut bacteria has emerged as an increasingly interesting function. About two thirds of drugs could be metabolized by at least one strain in the GM [15]. Compound modifications by gut microorganisms via enzymatic transformation alter bioavailability, bioactivity and toxicity, potentially leading to intestinal and systemic pharmacological effects [16]. Chemical transformation by microorganisms (biotransformation) is not the only mechanism implied. Bioaccumulation, consisting of bacteria storing the drug intracellularly without chemically modifying it, has also recently emerged as a common mechanism that alters drug availability with potential implications for pharmacokinetics, side effects and drug responses [17]. The GM could therefore influence an individual’s response to a specific drug. However, the interaction between drugs and the GM is bidirectional, given that drugs could influence microbial composition. Cancer patients are commonly at high risk of infectious complications making antimicrobial therapy an essential component of their management. Numerous findings exist about the impact of antimicrobial drugs on intestinal ecosystem [18]. More recently, scientific interest has increased about the relationship between GM and other drugs that could shape microbial composition [16]. Some of these drugs are routinely used in the oncological setting, including chemotherapeutic agents, immunosuppressive agents, steroids, protonic pump inhibitors and biliary acids [19,20]. The interaction between these drugs and the GM is one of the key components of the complex interplay between the intestinal ecosystem and the host during anticancer therapies. This bidirectional relationship between GM and cancer presents with distinctive features in pediatric patients, considering the rapidly and continuously evolving GM community, the strong impact of complications in growing subjects and the different subsets of diseases that require specific therapies [21,22]. Therefore, the pediatric setting deserves a specific focus when addressing pharmacomicrobiomics. In this paper, we aim to provide a complete overview on the bidirectional relationship between GM and drugs commonly used in the clinical practice in pediatric patients with cancer (Figure 1).

The main studies addressing the relationship between drugs commonly used in pediatric patients and gut microbiota are summarized in Table 1.

## 2. Antibiotics

**Figure 2:**
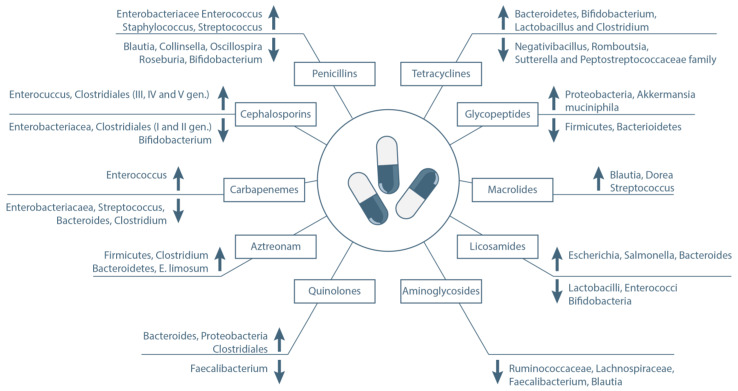
Impact of main antibacterial molecules on clinically relevant gut bacterial species.

The advances in the understanding of the role of GM in human health have changed our views on antibiotic use in the last few years, particularly during chemotherapy or in immunocompromised patients. Antibiotic use in these patients could represent a double-edged sword. Other than the anti-infective function, antibiotics are known to be strong negative modulators of the GM [50]. Their detrimental role was already known, as their use is associated with metabolic alterations and with the emergence of pathogenic strains, such as *C. Difficile* [51]. However, the emerging field of microbiota research has revealed deep and complex antibiotic-related modifications in the gut flora that result in various positive or detrimental clinical effects [50,52]. This seems to be mediated by a selective pressure by the antibiotics on the GM resulting in different patterns of modification depending on the antibacterial activity spectrum. Moreover, it has been demonstrated that antibiotic-driven modifications of the GM can last long after treatment with relevant later consequences [53]. Considering the high rate of antibiotics administrated during anticancer therapies, the effect on GM is prominent in these and should be considered in clinical decision algorithms [54]. While the topic should deserve a specific focus considering the extent of the available literature (systematic review can be found in reference [55]), we herein report the effect of the main antibacterial molecules on GM with a particular focus on bacteria with a proven effect on outcome for cancer patients (Figure 2).

### 2.1. β-Lactams

β-lactams are frequently used as first line antibiotic therapy for febrile neutropenia in children with cancer [56,57]. Recent ECIL8 guidelines recommend the use of an antipseudomonal non-carbapenem β-lactam/β-lactamase inhibitor combination for clinically stable patients, whereas carbapenems are reserved for clinically unstable conditions [58]. β-lactams are characterized by a wide antibacterial spectrum, appropriate for empirical therapies. Following their use, changes in microbial composition persist long after the end of the therapy. A long term alteration has been reported that lasts up to 12 weeks after treatment, with an incomplete restoration of microbial composition and emergence of antibiotic-resistant strains [59]. However, other authors reported complete recovery of GM and resistome composition at baseline levels one week after a short-term antibiotic course [60]. All β-lactams have an effect of modification in the GM in terms of overall α and β diversity and at a taxonomic scale, but different types present particular effects. β-lactams have generally been reported to both increase and decrease the relative abundance of different strains, even if the decrease is more common [55]. Amoxicillin has been associated with an increased abundance of the family of *Enterobacteriacee* [61] and an increase of pathogenic genera such as *Enterococcus*, *Staphylococcus* and *Streptococcus* [62]. In contrast, other health promoting genera have been reported to be reduced in abundance, namely, *Blautia*, *Collinsella*, *Oscillospira* and *Roseburia* [63]. Some studies also reported a small increase in *Clostridiales clostridium* [64], an order known to be associated with positive clinical outcomes in an onco-hematological setting, as a producer of short chain fatty acids (SCFA), such as butyrate [65]. One study directly addressed the effect of penicillin administration on SCFA and did not find any changes in fecal concentrations during treatment [66].

Cephalosporins are broadly used in pediatric patients as well, often as the first line treatment for neutropenic fever [57,67]. Most studies reported a decrease of *Enterobacteriacea* and an increase of *Enterococcus* following their administration [68]. *Enterococcus* has been associated with negative clinical outcomes both in leukemia and in HSCT patients [5,69]. Interestingly, the effect of cephalosporin on *Clostridiales* depends on the generation of the antibiotic. First and second generation cephalosporins have been associated with a decrease, while third, fourth and fifth generation with an increase of *Clostridiales* [70]. SCFA have been measured in one study showing a decrease after ceftriaxone administration [71]. The genus of Bifidobacterium is commonly decreased after treatment with penicillins and cephalosporins [72]. This represent an important genus in pediatric cancer patients, being far more present in children rather than in adults [8], and alterations in Bifidobacterium abundances can last up to 6 weeks following treatment [73]. Cefepime is frequently used in this setting and was specifically addressed in several studies [74]. In a cohort of HSCT recipients, cefepime was found not to exert an effect on intestinal diversity suggesting a potential protective role [75]. From a compositional point of view, cefepime has been associated with a decrease in *E. coli* and bifidobacteria abundances and an increase in *Bacteroides* spp. and *C. difficile* [76].

Carbapenems represent a β-lactams subclass with a narrower antimicrobial spectrum, usually used as second line therapy. Their use has been associated with a decrease in the abundance of *Enterobacteriacaea*, *Streptococcus* spp., *Bacteroides* spp., *Clostridium* spp. and an increased abundance of *Enterococcus* spp. [77].

Among monobactams, aztreonam is the most frequently used. Recently its use has been associated with positive outcome in HSCT recipients, suggesting a little suppressive effect on GM [51]. Two studies addressed the effect of aztreonam on GM showing an increase in *Firmicutes*, *Clostridium*, *Bacteroidetes* and *E. limosum* [78,79]. Interestingly, this latter has been associated with a reduced incidence of relapse after HSCT [80].

### 2.2. Quinolones

Quinolones inhibit the ligase activity of the type II topoisomerases, DNA gyrase and topoisomerase IV. They are used mainly as prophylactic agents during chemotherapy and HSCT [81,82]. They are also used as step-down therapy following episodes of febrile neutropenia [83]. The effect of quinolones on gut microbiota is variable, with reports of increase in the Bacteroides, *Proteobacteria* family and members of the *Clostridiales* order as well as decrease in *Faecalibacterium* [84,85,86]. Quinolone-induced modifications can last for a variable period, from 1 month up to one year [84,85].

### 2.3. Tetracyclines

Tetracyclines inhibit protein synthesis by binding to the 30S and 50S subunit of microbial ribosomes. Their use in pediatric patients is quite limited considering their negative effect of dental discoloration; however, they can be used in particular situation in pediatric cancer patients [87]. Patients receiving tetracyclines presents an increase of Bacteroidetes and a decrease of *Bifidobacterium*, *Lactobacillus* and *Clostridium* [88]. Moreover, a decrease of *Negativibacillus*, *Romboutsia*, *Sutterella* and *Peptostreptococcaceae* family was reported [63].

### 2.4. Glycopeptides

Glycopeptides are characterized by the ability to inhibit synthesis of the cell wall in susceptible microbes by inhibiting peptidoglycan synthesis. They are particularly used in neutropenic patients when Gram-positive bacteria are suspected. Glycopeptides are associated with a reduced fecal microbial diversity, with a decrease of Gram-positive bacteria, particularly Firmicutes, compensated by Gram-negative bacteria, mainly Proteobacteria [85], confirmed also in murine models [89,90]. In other studies the depletion of Gram-positive bacteria has been replaced by *Akkermansia muciniphila* [91], a strain associated with longer neutropenic fever in pediatric allogeneic HSCT (allo-HSCT) recipients [92] and worse outcome in cancer patients [52].

### 2.5. Macrolides

Macrolide antibiotics are protein synthesis inhibitors, binding reversibly to the P site on the 50S subunit of the bacterial ribosome. They are mainly used for the treatment of intracellular bacteria. The effect of macrolide on GM is variable, impacting the abundance of many taxa and reducing the α-diversity [55,93]. Interestingly, its use is often associated with colonization of Gram-positive bacteria. In particular, the bacterial depletion mediated by macrolide has been reported to be occupied by Gram-positive anaerobes, predominantly Blautia and Dorea, intestinal commensal organisms within the bacterial class Clostridia, particularly important in pediatric cancer patients in which Clostridia has been associated with lower complications rates [3]. These modifications seem to be persistent after the antibiotic discontinuation [94]. Macrolides have also been associated with increased abundances of Streptococcus [95], associated with higher infectious complications in pediatric patients [96]. Interestingly, recent evidence showed that the GM of pediatric patients prior to allo-HSCT is enriched with genes coding for drug resistance to macrolides [14].

### 2.6. Other Antibiotics

Other less studied antibiotics have been associated to different extents with microbiota modifications. Licosamides in healthy volunteers affected the GM substantially, resulting in a reduction of Lactobacilli, Enterococci and Bifidobacteria [85]. In hospitalized patients, clindamycin treatment was associated with increases in *Escherichia* spp., *Salmonella* spp. and *Bacteroides* spp. [72]. Aminoglycosides treatment in healthy volunteers has been associated with a decrease in *Ruminococcaceae*, *Lachnospiraceae*, *Faecalibacterium* spp. and *Blautia* spp. [97].

## 3. Chemotherapeutic Agents

The relationship between GM and chemotherapy drug metabolism represent a rapidly expanding field of research [98]. The reciprocal targeted modulation has demonstrated to affect both efficacy and adverse drug reactions of several drugs commonly used as part of pediatric antineoplastic treatments.

### 3.1. Irinotecan

Irinotecan is an antineoplastic agent used primarily for the treatment of soft tissue sarcomas, bone tumors and neuroblastoma in children [99,100]. It is administered as a prodrug, CPT-11, which requires enzymatic conversion by carboxylesterase. SN-38 is the active metabolite that acts as a topoisomerase I inhibitor, causing single-strand DNA breaks and ultimately cell death [101]. The SN-38 is then glucuronidated in the liver by UDP-glucuronosyltransferase into its less toxic derivate, SN-38-G, and later transported in the bile and excreted to the intestinal lumen. Some symbiotic species of GM can produce β-glucuronidase that reverts SN-38-G to its active and more toxic form, SN-38, increasing irinotecan intestinal toxicity. These bacteria include *Escherichia coli*, *Bacteroides vulgatus*, and *Clostridium ramosum* [23,102]. The main adverse effect associated with irinotecan is diarrhea, which can be acute or delayed [103]. Delayed-type diarrhea can be severe and potentially dose-limiting [104]. The exact mechanism is still unknown but probably directly mediated by high concentration of intraluminal SN-38 [105]. Interestingly, selective inhibition of β-glucuronidase activity has proven successful in alleviating intestinal toxicity in mice [102]. Furthermore, Irinotecan treatment itself can modify the host GM composition, increasing the number of β-glucuronidases-expressing species, such as *E. coli*, *Staphylococcus* spp., and *Clostridium* spp. [24], which could potentially amplify this effect. Considering the role of GM components on irinotecan metabolite-induced diarrhea, the potential utility of antibiotics coadministration with irinotecan has been studied, with positive results. The administration penicillin/streptomycin, to irinotecan-treated rats resulted in decreased levels of SN-38 in the feces and reduced diarrhea [105]. Although some early studies suggested a role of neomycin in reducing irinotecan-induced delayed-diarrhea [106], some later evidence mitigated these results [107]. Despite its possible utility, the use of concomitant prophylactic antibiotics with chemotherapy is controversial, due to possible emergence of antibiotic resistance and impact on GM composition. More specific strategies to target β-glucuronidase activity have been investigated, including the “old” drugs such as Amoxapine to inhibit β-glucuronidases [108]. 3D X-ray crystallographic data are also under investigation in order to rationally design a β-glucuronidase inhibitor [102]. More recent pharmacological compounds have been tested with positive results [109], but their application in clinical practice has not been yet validated.

### 3.2. Cyclophosphamide

Cyclophosphamide (CP) is an alkylating agent with immunosuppressant and anticancer effects. It is widely used in the treatment of immune dysregulatory conditions and malignancies. CP is a prodrug, which requires metabolic activation. Its active form exerts its therapeutic activity through several mechanisms. The main active metabolite is phosphoramide mustard, which forms irreversible DNA crosslinks both between and within DNA strands at guanine N-7 positions, leading to cell apoptosis [110]. CP also promotes the differentiation of antitumor Th1 and Th17 cells [111], depletes oncogenic regulatory T-cells and induces the production of pro-apoptotic cytokines, promoting immune-driven cancer cell death [112]. Viaud et al. demonstrated that germ-free mice, treated with broad-spectrum antibiotics, showed a significantly reduced anticancer response after CP administration [25]. CP administration resulted in increased IL-17 levels in specific-pathogen-free (SPF) mice against germ-free (GF) mice. The translocation of specific Gram-positive bacteria (such as *Lactobacillus johnsonii* and *Enterococcus hirae*) from the intestine to secondary lymphoid organs was critical for the differentiation of CD4+ T cells into Th1 and Th17 cells. Furthermore, CP and vancomycin cotreatment resulted in overall worse anticancer response in their animal model, altering CTX-induced Th17 differentiation, which is mandatory for the tumoricidal activity of chemotherapy [25]. More recent studies confirmed the synergic interplay between specific bacterial species (such as *Enterococcus hirae* and *Barnesiella intestinihominis*) and CP, facilitating its antitumoral activity [113]. Moreover, CP treatment has an impact on the GM composition. When comparing the GM of both CP-treated and CP-naïve mice, the treatment reduced fecal bacterial diversity, increased the proportion of Firmicutes, and decreased the proportion of *Bacteroidetes bacteria* [26]. Recent studies investigated the potential modulation of various polysaccharides compounds on the effects of CP on immune modulation, intestinal permeability, and microbial communities in the mouse with mixed results [26,114,115,116,117].

### 3.3. L-Asparaginase

L-Asparaginase (ASNase) is a critical chemotherapeutic compound of many acute lymphoblastic leukemia and lymphoma protocols [118,119]. ASNase breaks extracellular asparagine, an amino acid required for protein assembly of leukemic cells. ASNase treatment is often accompanied by severe adverse reactions. Since ASNase is a non-self-protein, antibodies may develop against it which can lead to hypersensitivity reactions occurring in 25% of patients and undetected inactivation of ASNase, which correlates with a poor response to treatment [120]. Two ASNase formulations are currently available, polyethylene-glycolated (PEG) form of the *E. coli* ASNase (PEG-ASNase) and Erwinia crysanthemi-derived ASPase [121]. Endogenous GM strains, such as *Salmonella* or *Shigella flexneri*, naturally produce the L-asparaginase periplasmic enzymes that are similar to PEG-ASNase for 96 and 99.1% of their molecular structure, respectively [118,122]. A recent study demonstrated that specific GM communities were associated with different ASNase activity levels in treated children. Escherichia predominated in the decreased-activity community while Bacteroides and Streptococcus predominated in the increased-activity community [27]. Furthermore, microbial ASNS was significantly negatively correlated with change in serum ASNase activity, although the mechanism remains unknown [27].

### 3.4. Other Chemotherapeutic Drugs

Other chemotherapeutic agents have shown interaction with GM composition. For example, the alkylating agent Melphalan has been found to be metabolized by “super metabolizer” bacterial strains such as *Bacteroides dorei* and *Clostridium* spp. [15]. In recent years, evidence emerged about the inactivation through deglycosylation of the widely adopted anthracycline Doxorubicin mediated by specific gut bacterial strains [123,124,125], but further studies are needed to confirm its clinical relevance in vivo.

## 4. Anti-Programmed Cell Death Proteins

In recent years, immunotherapies and immune checkpoint inhibitors emerged as alternatives or complementary to conventional chemotherapy in the treatment of various malignancies. The interaction between PD-L1 and PD-1 is intended as an immune checkpoint in various physiological situations, such as immune tolerance in pregnancy, to prevent self-rejection and minimize the inflammatory response. However, during many carcinogenic processes, the activation of the PD-L1/PD-1 signaling cascade results in decreased T-cell activation, leading to a reduced anticancer immune response [126,127]. A wide variety of anti-PD-1/PD-L1 antibodies have been developed to treat multiple malignancies including Hodgkin lymphoma, sarcomas, melanoma, and small cell lung cancers. However, the rise of anti-PD-1 therapy is accompanied by significant variability in patient response to these inhibitors. Given the recent understanding of the complex interaction between the GM and the immune response, researchers examined the interaction of the host gut microbial community with anti-PD-1/PD-L1 inhibitors. Sivan and colleagues firstly showed that commensal Bifidobacteria have a positive association with antitumor T-cell response and Bifidobacterium-treated mice showed a significant improvement in tumor control [128]. Studies on human stool samples from metastatic melanoma patients were also conducted. Through the integration of 16S ribosomal RNA gene sequencing, metagenomic shotgun sequencing, and quantitative polymerase chain reaction, a significant association between commensal microbial composition and clinical response was demonstrated, with *Bifidobacterium longum*, *Collinsella aerofaciens*, and *Enterococcus faecium* more abundant in stool samples of responders [129]. Moreover, the anti-PD-1 responders had presented greater levels of α-diversity and higher proportions of *Ruminococcaceae*, *Faecalibacterium*, and *Bifidobacterium* species [28]. Microbiome composition alters anti-PD1 response, with *Akkermansia*, *Ruminococcus* spp., *Alistipes* spp., and *Eubacterium* spp. being more represented in drug responders, while under-representation was found for *Bifidobacterium adolescentis*, *B. long* and *Parabacteroids distasonis* in drug responders [29]. Furthermore, avoiding antibiotics during anti-PD-1 treatment could increase patients’ positive responses from 25% up to 40%. In the setting of advanced melanoma, tumor cells may become resistant to anti-PD-1 agents. Davar et al. proposed fecal microbiota transplantation (FMT) as a strategy to promote a positive and durable gut microbiome perturbation. Responders exhibited an increased abundance of taxa that were previously shown to be associated with response to anti-PD-1, increased CD8+ T cell activation, and decreased frequency of interleukin-8-expressing myeloid cells. This reprogrammed tumor microenvironment did overcome resistance to anti-PD-1 in this subset of PD-1 advanced melanoma [7]. Notably, Tanoue et al. performed a phase 1/2 trial with an oral microbial product (VE800) that contains 11 clonal commensal bacterial strains from healthy human donor feces and that is capable of robustly inducing interferon-γ-producing CD8 T cells in the intestine. The study showed that colonization of mice with the 11-strain mixture enhances both host resistance against Listeria monocytogenes infection and the therapeutic efficacy of immune checkpoint inhibitors in syngeneic tumor models [130]. The impact of dietary habits and commercially available probiotic supplements on fecal microbiota profiles of patients treated with anti-PD-1 for melanoma, has also been investigated. 128 patients with sufficient dietary fiber intake showed a significant improvement in progression free survival compared to insufficient fiber intake, with no impact of probiotic administration [131]. Other microbiome-modifying interventions have been proposed to enhance immune checkpoint inhibitor antitumor activity, such as natural polyphenols [132] and ketogenic diet [133], but more evidence is needed to implement those interventions in routine clinical practice [22]. Similarly, anti-CTLA-4 antibodies are used as checkpoint inhibitors in many malignancies. Notably, GM composition seems to present a strong influence also on the efficacy of this form of immunotherapy [134]. These findings suggest that the GM has a significant impact on the efficacy of immune checkpoint inhibitors in a variety of malignancy models [135,136,137], especially in specific subsets of patients that show low response to this treatment.

## 5. Immunosuppressive Agents

The interaction between GM and host’s immune system is bidirectional. Microbiota plays a major role in the development and regulation of the immune system, while the immune system controls the microbiota through the production and secretion of antimicrobial peptides and secretory IgA [40,138].

### 5.1. Cyclosporine

Cyclosporine (CSA) is a calcineurin inhibitor able to bind to immunophilins called cyclophilins, leading to an increased cyclophilin affinity to calcineurin, a calmodulin-activated serine phosphatase that associates with NFAT (nuclear factor of activated T cells) and initiates events involved in T-cell activation. The complex cyclosporine-cyclophillin binds and inhibits calcineurin, blocking the synthesis of proinflammatory cytokines and interrupting the downstream sequence of events leading to rejection [139]. O’Reilly et al. noticed no significant difference in α or β diversity with CSA use. CSA decreases the viability of B. distasonis, but it does not affect Lactobacillus or Bifidobacterium species [31]. On the other hand, Junjun Jia et al. demonstrated in rats an improved diversity of the intestinal microbiota and a richness of species, with an enrichment of Enterobacteriaceae and a decrease of F. prausnitzii and Clostridium clusters I and XIV, with CSA use [30].

### 5.2. Tacrolimus

Similar to CSA, tacrolimus is also a calcineurin inhibitor, that binds to different immunophilins called FK-binding proteins, with a block of T-cell activation [139]. Tacrolimus in rats decreases microbial diversity and increases the Firmicutes/Bacteroidetes ratio [34]. Tacrolimus does not change the bacterial richness and diversity of GM, without difference at the phylum level, but with an increase in the relative abundance of Allobaculum, Bacteroides and Lactobacillus [32]. In rats, a decreased abundance of *Mollicutes*, *Micrococcaceae*, *Actinomycetales*, *Roseburia*, *Oscillospira*, *Rothia* and *Staphylococcus* and increased *A. muciniphila* was also observed [33]. Tacrolimus triggers a gut dysbiosis that is analogous to that observed in metabolic diseases, with increased Firmicutes/Bacteroides ratio [138]. Interestingly, in a rat model, Jiang at al showed that an intermediate dose was associated with an increase in beneficial bacteria (Bifidobacterium, *F. prausnitzii*) and a decrease in less beneficial bacteria (Enterobacteriaceae, Bacteroides-Prevotella). On the other hand, lower and higher doses were associated with increased abundance of Enterobacteriaceae and decrease of Bifidobacterium and *F. prausnitzii* [35]. Patients who required high doses of tacrolimus harbored a higher relative abundance of *F. prausnitzii* in their GM, and *F. prausnitzii* abundance at 1 week after kidney transplant was positively correlated with future dosing of tacrolimus at 1 month [140]. Some gut bacteria, such as *F. prausnitzii* or Clostridiales, seem to transform tacrolimus into a 15-fold less active metabolite in vitro.

### 5.3. Other Immunosuppressive Drugs

Mycophenolate mofetil (MMF) is a pro-drug that is converted into the active metabolite mycophenolic acid (MPA), which inhibits inosine monophosphate dehydrogenase and suppresses the proliferation of T and B lymphocytes [36]. MMF causes GI toxicity in 30–50% of patients, ranging from nausea, vomiting, diarrhea, abdominal pain to a colitis that resembles IBD [36,138]. In mice, MMF treatment causes a decrease in microbiota richness, with a reduction in Bacteroidetes and Verrucomicrobia phyla and in the genera Akkermansia, Parabacteroides and Clostridium, and increased Proteobacteria (mainly Escherichia/Shigella), Deferribacters and Firmicutes. These changes result in a shift of the microbiota toward one with greater pathogenic potential [36]. MMF has a narrow therapeutic index and blood concentrations of MPA are highly variable, probably also depending on MPA enterohepatic recirculation (EHR), with patients with higher HER having a better immunosuppression but also more concentration-dependent toxicities. Thus Saqr et al. studied how bacteria influence EHR, showing that MPA EHR is positively correlated with *B. vulgatus* and *B. thetaiotaomicron* and negatively correlated with *Blautia hydrogenotrophica*.

Rapamycin, also known as sirolimus, inhibits mTOR, a protein kinase that regulates cell growth, proliferation and survival, thus interfering with lymphocyte proliferation [138]. Bhat et al. showed in a rat model that bacterial diversity is significantly decreased with the use of rapamycin, with decreased *Roseburia*, *Oscillospira*, *Mollicutes*, *Rothia*, *Micrococcaceae*, *Acninomycetales* and *Staphylococcus*. Decreased Turicibacter, unclassified *Marinilabiliaceae*, *Alloprevotella*, unclassified *Porphyromonadaceae*, *Ruminococcus*, *Bifidobacterium*, *Marvinbryantia*, *Helicobacter* and *Coprobacillus* in rapamycim-treated mice, and increased *Ruminococcus* were also reported [37].

Alemtuzumab is a monoclonal antibody that targets CD52 expressed on T and B lymphocytes, natural killer cells and monocytes, inducing rapid depletion of T cells from peripheral blood. In a monkey model, Li et al. showed that alemtuzumab treatment causes reduced *Lactobacillales*, and increased *Enterobacteriales* and *Prevotella*. In the GM, they also noticed an increase in the *Clostridiales* order and a decrease in *Faecalibacterium* genus [38].

## 6. Steroids

Glucocorticoids are the mainstays in the treatment of inflammatory and autoimmune pathologies, and they are used as immunosuppressants following organ transplantation and as lympholytics in chemotherapeutic regimens. Glucocorticoids reduce inflammation by suppressing pro-inflammatory cytokine expression through inhibition and upregulation of gene transcription [141]. In a mouse model, glucocorticoids decreased bacterial richness and diversity, reduced relative abundance of *Firmicutes*, *Bacteroides*, *Actinobacteria*, alfa and gamma *Proteobacteria*, and decreased *Clostridiales* and *Lactobacillus*. On the other hand, steroids increased abundance of *Proteobacteria*, closely related to a proinflammatory state [40,41,42]. A decrease of *Deferribacteres*, *Rikenella*, *Mucispirillum*, *Oscillospira* and *Bilophila*, and an increase in *Prevotella*, *Methanobrevibacter smithii* and *Anaerostipes* were also reported [39,42,43]. In the gut, *Clostridium scindens* converts endogenous glucocorticoids into androgens [142]. It has been demonstrated that dexamethasone is metabolized into androgens by *Clostridium scindens* in vivo, with potential implications also for other steroids [15].

## 7. Protonic Pump Inhibitors

Protonic pump inhibitors (PPIs) are among the most commonly used drugs worldwide [16,143]. PPIs are prodrugs that need to be activated by addition of two protons and, once they are activated, they can inactivate the proton pump by binding to the H+-K+-ATPase that normally creates a 1 million-fold gradient in H+ concentration from inside the parietal cell to the gastric lumen in return for inward transport of K+ [143]. A first report on 20 children treated with PPI found no significant changes in overall number of species-level taxonomy categories but with an increase in the phylum *Firmicutes* in some subgroups [48]. Other studies demonstrated that PPI use is associated with an altered composition of the GM, with an increase in the *Lactobacillales* order and in particular the family *Streptococcaceae*, which has been associated with increased risk for *C. difficile* infection (CDI) [43,44,45,46,144]. A strong tendency of a reduction of *Faecalibacterium*, which is known to possess anti-inflammatory properties, was also reported [47]. Characteristic of the gut microbiome of PPI users are species highly prevalent in the oral microbiome, such as *Streptococcus parasanguinis* [43]. Higher dosages are associated with larger microbial changes and functional changes, such as an increase of fatty acid and lipid biosynthesis, fermentation NAD metabolism and biosynthesis of L-arginine and purine deoxyribonucleoside degradation [43]. These changes could result from downward movement of upper tract commensals due to removal of the gastric acid barrier by PPI, causing an “oralisation” of the GM in PPI users. However, PPI also have a direct effect on the GM, potentially generated through binding of PPIs to bacterial H+/K+ ATPases [16,145]. A trend toward reduced diversity was also reported in some reports, even if not always significant [44,45]. Other studies showed no major changes in diversity [46,47,48].

## 8. Ursodeoxycholic Acid

Ursodeoxycholic acid is a naturally occurring bile acid that is used to treat a variety of hepatic and gastrointestinal diseases and also specifically in HSCT, for prevention of hepatic complications. Bile acids have recently emerged as important regulators of the intestinal microbiota; thus, it is interesting to see how ursodiol modifies the microbiota, but not many studies have investigated this [146]. Studies conducted on patients with primary biliary cholangitis show that UDCA causes an increased abundance of the *Enterobacteriaceae* family [49]. Pearson et al. showed in patients with a story of colorectal adenomans that UDCA causes a shift in microbial community composition, with an increase in species of *Streptococcus*, *Escherichia* and *Bilophila* and decrease in *Fusobacterium*, and in particular an overrepresentation of *Faecalibacterium prausnitzii* and an underrepresentation of *Ruminococcus gnavus* [20]. In mice, ursodeoxycholic acid increases several key bile acid species, that in turn alter directly or indirectly the gut microbial composition [147].

## 9. Conclusions

We provided an outline of the interaction between commonly used drugs in pediatric oncology and the GM. Pharmacomicrobiomics is an emerging field of study that could change the outlook of research both regarding pharmacology and microbiome studies. The intestinal flora composition is an issue to be considered in the individual variability of pharmacokinetics, response to therapy and adverse event rate, other than the usual studied genetic polymorphism. The impact of drugs on the GM should be taken into consideration in the future among the factors that can influence trial design and drug prescribing, with potential implications related to GM modulation [148]. In the near future, testing of the microbiome may provide a tool to help guide initial dose selection and dose adjustments of selected drugs, such as in the case of MMF. Moreover, it could help in the personalized follow up of patients at higher risk of treatment-related toxicity or treatment failure. Thanks to this field of research, targeting the microbiome could be an interesting future perspective in order to reduce the number of poor responders or patients experiencing severe adverse events in selected cases. A deeper understanding of the biological mechanism underpinning this complex interplay is needed before translating the presented findings to clinical research. Moreover, dissecting the effect of single drugs in the human setting is complex, because in oncology different molecules are often administered together and pharmacological interactions are a key component in the management of pediatric cancer patients. A better knowledge on the impact of GM on drug metabolism could lead to fascinating results, potentially translating to clinical practice.

## Figures and Tables

**Table 1:** Studies addressing the relationship between drugs commonly used in pediatric patients and gut microbiota. ↑, increased; ↓, decreased.

Drug Name	Year	First Author	Setting	Sample Size	Interaction with Gut Microbiome	Ref
**Irinotecan (CPT-11)**	2015	Wallace, BD	Preclinical	/	Intestine bacteria producing β-glucuronidase can convert non-toxic CPT-11 metabolite (SN-38-G) to toxic metabolite (SN-38), causing diarrhea.	[23]
**Irinotecan (CPT-11)**	2008	Stringer, AM	Preclinical, rats	/	↑ number of β-glucuronidases-expressing species.	[24]
**Cyclophosphamide**	2013	Viaud, S	Preclinical	/	Translocation of specific Gram-positive bacteria from the intestine to secondary lymphoid organs was critical for the differentiation of CD4+ T cells into Th1 and Th17 cells.	[25]
**Cyclophosphamide**	2015	Xu, X	Preclinical	/	↑ *Firmicutes*, ↓ *Bacteroidetes.*	[26]
**L-asparaginase**	2021	Dunn, KA	Pediatric ALL	12 patients	↑ *Escherichia* in the community if decreased-activity, ↑ *Bacteroides* and *Streptococcus* in the community if increased-activity.	[27]
**Anti-PD1**	2018	Gopalakrishnan, V	Adults, melanoma	112 patients	↑ α-diversity of responders to anti-PD1 therapy. Higher proportion of *Ruminococcaceae*, *Faecalibacterium*, and *Bifidobacterium* spp. reported in responders.	[28]
**Anti-PD1**	2018	Routy, B	Mice, Adults	Mice, 249 treated	↑ *Akkermansia*, *Ruminococcus spp.*, *Alistipes spp.*, and *Eubacterium spp* in responders. ↓ *Bifidobacterium adolescentis*, *B. longum*, and *Parabacteroids distasonis* in responders.	[29]
**Cyclosporine**	2019	Jia et al.	Preclinical	8 treated	↑ gut microbial richness, *Enterobacteriaceae*. ↓ *F. prausnitzii*, *Clostridium* clusters I and XIV.	[30]
**Cyclosporine**	2020	O Reilly et al.	Adults	6 ex vivo, 8 in vivo	No significant α and β diversity before and after treatment.	[31]
**Tacrolimus**	2017	Zhang et al.	Mice	8 treated	No change in bacterial richness and diversity. ↑ genera *Allobaculum*, *Bacteroides* and *Lactobacillus*. ↓ *Clostridiales*, *Ruminococcaceae*, *Rikenella*, *Ruminococcaceae* and *Oscillospira.*	[32]
**Tacrolimus**	2017	Bhat et al.	Mice	5 treated	↓ *Mollicutes*, *Micrococcaceae*, *Actinomycetales*, *Roseburia*, *Oscillospira*, *Rothia* and *Staphylococcus*. ↑ *A. muciniphila.*	[33]
**Tacrolimus**	2018	Toral et al.	Mice	8 treated	↓ microbial diversity. ↑ *Firmicutes/Bacteroidetes* ratio.	[34]
**Tacrolimus**	2018	Jiang et al.	Mice	8 high dosage, 8 medium dosage, 8 low dosage	Intermediate dose: ↑ *Bifidobacterium*, *Faecalibacterium prausnitzii* ↓ less *Enterobacteriaceae*, *Bacteroides-Prevotella* Low and high doses: ↑ *Enterobacteriaceae* ↓ *Bifidobacterium*, *Faecalibacterium prausnitzii.*	[35]
**MMF**	2018	Flannigan et al.	Mice	9 treated	↓ overall diversity ↑ Proteobacteria (Escherichia/Shigella), Deferribacteres, Firmicutes ↓ Bacteroidetes and Verrucomicrobia phyla, Akkermansia, Parabacteroides and Clostridium genera.	[36]
**Rapamycin**	2017	Bhat et al.,	Mice	5 treated	↓ bacterial diversity. ↓ *Roseburia*, *Oscillospira*, *Mollicutes*, *Rothia*, *Micrococcaceae*, *Acninomycetales* and *Staphylococcus.*	[33]
**Rapamycin**	2016	Jung et al.	Mice	5 treated	↓ *Turicibacter*, unclassified *Marinilabiliaceae*, *Alloprevotella*. ↑ *Ruminococcus*.	[37]
**Alemtuzumab**	2013	Li et al.	Monkeys	15 treated	↑ Enterobacteriales order and Prevotella genus. ↓ Lactobacillales order.	[38]
**Steroids**	2014	Lee et al.	Humans	4 treated	↓ *Clostridiales* ↑ *Erysipelotrichales.*	[39]
**Steroids**	2016	Tourret et al.	Mice	8–10 treated	↑ *Firmicutes/Bacteroidetes* ratio ↓ *Clostridium sensu stricto.*	[40]
**Steroids**	2017	Wu et al.	Mice	30 lower dose, 30 higher dose	↓ bacterial richness and diversity. ↓ *Firmicutes*, *Bacteroides*, *Actinobacteria*, α and γ *Proteobacteria*, *Clostridiales* and *Lactobacillus.* ↑ Proteobacteria.	[41]
**Steroids**	2019	He et al.	Mice	10 treated	↓ *Proteobacteria*, *Deferribacteres*, *Rikenella*, *Mucispirillum*, *Oscillospira* and *Bilophila.* ↑ *Prevotella* and *Anaerostipes.*	[42]
**Steroids**	2020	Vich Vila et al.	Adults	17 treated	↑ *Methanobrevibacter smithii* and *Streptococcus salivarius*.	[43]
**PPI**	2016	Jackson et al.	Adults	1827	↓ diversity in PPI users. ↑ *Lactobacillales* order, families *Micrococcaceae* and *Streptococcaceae*, genera *Rothia* and *Streptococcus*, species *Rothia mucilaginosa* and *Streptococcus anginosus.* ↓ families *Erysipelotrichaceae*, *Lachnospiraceae*, *Ruminococcaceae*, genera *Firmicutes*, species *Erysipelotrichales* and *Clostridiales.*	[44]
**PPI**	2015	Imhann et al.	Adults	99 treated	↓ species richness and ↓ Shannon diversity, although not significant. ↑ *Gammaproteobacteria* class, *Actinomycetales* order, families *Streptococcaceae* and *Micrococcaceae*, genera *Rothia*, *Streptococcus* and *Veilonella*, species *Lactobacillus salivarius.*	[45]
**PPI**	2015	Freedberg et al.	Adults	12 treated	No changes in diversity. ↑ families *Enterococcaceae*, *Streptococcaceae*, *Micrococcaceae* and *Staphylococcaceae*. ↓ *Clostridiales.*	[46]
**PPI**	2015	Tsuda et al.	Adults	18 treated	No changes in α diversity, increased β diversity. ↓ genus *Faecalibacterium.*	[47]
**PPI**	2020	Vich Vila et al.	Adults	108 treated	↑ species *Veillonella parvula*, *Streptococcus salivarius*, *Streptococcus parasanguinis*, *Streptococcus vestibularis*, *Bifidobacterium dentium*, *Haemophilus parainfluenzae.*	[43]
**PPI**	2021	Simakachorn et al.	Pediatrics	20 treated	No significant change in α and β diversity. No change in total number of species-level taxonomy categories.	[48]
**UDCA**	2018	Pearson et al.	Adults	661 treated	No change in microbial richness. ↑ *Streptocuccus*, *Escherichia* and *Bilophila* spp., *Faecalibacterium prausnitzii*; ↓ *Fusobacterium* spp., *Ruminococcus gnavus.*	[20]
**UDCA**	2018	Tang et al.	Adults	60 treated	↑ *Enterobacteriaceae.*	[49]

## Data Availability

Not applicable.
